# Analysis of COVID-19 Guideline Quality and Change of Recommendations: A Systematic Review

**DOI:** 10.34133/2021/9806173

**Published:** 2021-07-22

**Authors:** Siya Zhao, Shuya Lu, Shouyuan Wu, Zijun Wang, Qiangqiang Guo, Qianling Shi, Hairong Zhang, Juanjuan Zhang, Hui Liu, Yunlan Liu, Xianzhuo Zhang, Ling Wang, Mengjuan Ren, Ping Wang, Hui Lan, Qi Zhou, Yajia Sun, Jin Cao, Qinyuan Li, Janne Estill, Joseph L. Mathew, Hyeong Sik Ahn, Myeong Soo Lee, Xiaohui Wang, Chenyan Zhou, Yaolong Chen

**Affiliations:** ^1^School of Public Health, Lanzhou University, Lanzhou, China; ^2^Institute of Health Data Science, Lanzhou University, Lanzhou, China; ^3^Department of Pediatric, Sichuan Provincial People's Hospital, University of Electronic Science and Technology of China, Chengdu, China; ^4^Chinese Academy of Sciences Sichuan Translational Medicine Research Hospital, Chengdu, China; ^5^Evidence-Based Medicine Center, School of Basic Medical Sciences, Lanzhou University, Lanzhou, China; ^6^The First School of Clinical Medicine, Lanzhou University, Lanzhou, China; ^7^National Clinical Research Center for Child Health and Disorders, Ministry of Education Key Laboratory of Child Development and Disorders, China International Science and Technology Cooperation Base of Child Development and Critical Disorders, Children's Hospital of Chongqing Medical University, Chongqing, China; ^8^Institute of Global Health, University of Geneva, Geneva, Switzerland; ^9^Institute of Mathematical Statistics and Actuarial Science, University of Bern, Bern, Switzerland; ^10^Advanced Pediatrics Centre, PGIMER Chandigarh, Chandigarh, India; ^11^Department of Preventive Medicine, Korea University, Seoul, Republic of Korea; ^12^Korea Cochrane Centre, Seoul, Republic of Korea; ^13^Evidence Based Medicine, Seoul, Republic of Korea; ^14^Korea University School of Medicine, Seoul, Republic of Korea; ^15^Korea Institute of Oriental Medicine, Daejeon, Republic of Korea; ^16^University of Science and Technology, Daejeon, Republic of Korea; ^17^London Southbank University, London, UK; ^18^Tianjin University of Traditional Chinese Medicine, Tianjin, China; ^19^WHO Collaborating Centre for Guideline Implementation and Knowledge Translation, Lanzhou, China; ^20^Guideline International Network Asia, China; ^21^Key Laboratory of Evidence Based Medicine and Knowledge Translation of Gansu Province, Lanzhou University, Lanzhou, China; ^22^Lanzhou University GRADE Center, China

## Abstract

**Background:**

Hundreds of coronavirus disease 2019 (COVID-19) clinical practice guidelines (CPGs) and expert consensus statements have been developed and published since the outbreak of the epidemic. However, these CPGs are of widely variable quality. So, this review is aimed at systematically evaluating the methodological and reporting qualities of COVID-19 CPGs, exploring factors that may influence their quality, and analyzing the change of recommendations in CPGs with evidence published.

**Methods:**

We searched five electronic databases and five websites from 1 January to 31 December 2020 to retrieve all COVID-19 CPGs. The assessment of the methodological and reporting qualities of CPGs was performed using the AGREE II instrument and RIGHT checklist. Recommendations and evidence used to make recommendations in the CPGs regarding some treatments for COVID-19 (remdesivir, glucocorticoids, hydroxychloroquine/chloroquine, interferon, and lopinavir-ritonavir) were also systematically assessed. And the statistical inference was performed to identify factors associated with the quality of CPGs.

**Results:**

We included a total of 92 COVID-19 CPGs developed by 19 countries. Overall, the RIGHT checklist reporting rate of COVID-19 CPGs was 33.0%, and the AGREE II domain score was 30.4%. The overall methodological and reporting qualities of COVID-19 CPGs gradually improved during the year 2020. Factors associated with high methodological and reporting qualities included the evidence-based development process, management of conflicts of interest, and use of established rating systems to assess the quality of evidence and strength of recommendations. The recommendations of only seven (7.6%) CPGs were informed by a systematic review of evidence, and these seven CPGs have relatively high methodological and reporting qualities, in which six of them fully meet the Institute of Medicine (IOM) criteria of guidelines. Besides, a rapid advice CPG developed by the World Health Organization (WHO) of the seven CPGs got the highest overall scores in methodological (72.8%) and reporting qualities (83.8%). Many CPGs covered the same clinical questions (it refers to the clinical questions on the effectiveness of treatments of remdesivir, glucocorticoids, hydroxychloroquine/chloroquine, interferon, and lopinavir-ritonavir in COVID-19 patients) and were published by different countries or organizations. Although randomized controlled trials and systematic reviews on the effectiveness of treatments of remdesivir, glucocorticoids, hydroxychloroquine/chloroquine, interferon, and lopinavir-ritonavir for patients with COVID-19 have been published, the recommendations on those treatments still varied greatly across COVID-19 CPGs published in different countries or regions, which may suggest that the CPGs do not make sufficient use of the latest evidence.

**Conclusions:**

Both the methodological and reporting qualities of COVID-19 CPGs increased over time, but there is still room for further improvement. The lack of effective use of available evidence and management of conflicts of interest were the main reasons for the low quality of the CPGs. The use of formal rating systems for the quality of evidence and strength of recommendations may help to improve the quality of CPGs in the context of the COVID-19 pandemic. During the pandemic, we suggest developing a living guideline of which recommendations are supported by a systematic review for it can facilitate the timely translation of the latest research findings to clinical practice. We also suggest that CPG developers should register the guidelines in a registration platform at the beginning for it can reduce duplication development of guidelines on the same clinical question, increase the transparency of the development process, and promote cooperation among guideline developers all over the world. Since the International Practice Guideline Registry Platform has been created, developers could register guidelines prospectively and internationally on this platform.

## 1. Introduction

COVID-19 has become a global pandemic. Up to 31 December 2020, there have been nearly 81.6 million confirmed cases and nearly 1.8 million deaths globally [1]. As yet, the WHO Rapid Evidence Appraisal for COVID-19 Therapies (REACT) Working Group has found that compared to patients receiving usual care or placebo, the 28-day all-cause mortality was lower in critically ill patients with COVID-19 receiving dexamethasone [2]. But the effective therapeutic options for COVID-19 patients are still limited, and those used are mostly supportive or palliative [3]. More than 5,000 clinical trials on COVID-19 have been ongoing or completed around the world [4]. Clinical practice guidelines (CPGs) that can timely translate the essential results of those studies into clinical practice should be developed and updated to help to respond to the COVID-19 pandemic.

COVID-19 CPGs have been developed by WHO and other developers after the outbreak. Among the published studies on COVID-19, CPGs and consensus statements accounted for one-fifth of the total [5]. However, previous studies have shown that CPGs developed early in the COVID-19 pandemic had methodological weaknesses [6–8]. And when there was no effective treatment against COVID-19, the treatment recommendations between some COVID-19 guidelines are highly consistent [9].

One year has passed since COVID-19 became known worldwide, and the number of COVID-19 CPGs continues to increase. But the methodological quality of some new COVID-19 CPGs, the overall reporting quality of COVID-19 CPGs, the quality change of COVID-19 CPGs in 2020, and the key factors associated with high methodological and reporting qualities of CPGs in the context of a global pandemic remain unknown. Furthermore, randomized controlled trials and systematic reviews on the effectiveness of treatments of remdesivir, glucocorticoids, hydroxychloroquine/chloroquine, interferon, and lopinavir-ritonavir for patients with COVID-19 have been published, and the results show that some of the treatments are effective for COVID-19 patients, and some are ineffective [2, 10–53]. But the consistency of recommendations on those treatments in COVID-19 CPGs remains unknown. Therefore, we aimed to analyze both the methodological and reporting qualities and their change of COVID-19 CPGs published from January 1, 2020, to December 31, 2020, to provide clinicians with a basis for appropriate selection of highest-quality guidelines and to provide guideline developers on how to develop high-quality CPGs during such a public health emergency globally. And we aimed to analyze the consistency of recommendations on treatments of remdesivir, glucocorticoids, hydroxychloroquine/chloroquine, interferon, and lopinavir-ritonavir in COVID-19 CPGs and to explore the change of recommendations with evidence published. In other words, we wanted to explore whether the treatment recommendations between COVID-19 guidelines are still highly consistent or not when there were effective treatments against COVID-19.

## 2. Methods

### 2.1. Search Strategy

The following five electronic databases were searched: MEDLINE (via PubMed), WHO COVID-19 database, China Biology Medicine disc (CBM), China National Knowledge Infrastructure (CNKI), and WanFang data. The following five websites were also searched for further potential CPGs: (1) the National Institute for Health and Care Excellence (NICE, https://www.nice.org.uk/); (2) the Scottish Intercollegiate Guidelines Network (SIGN, https://www.sign.ac.uk/); (3) the Guidelines International Network (GIN, https://g-i-n.net/); (4) the website of Centers for Disease Control and Prevention of the United States (https://www.cdc.gov/); and (5) the website of Centers for Disease Control and Prevention of China (http://www.chinacdc.cn/). Furthermore, we searched Google. We limited the search to CPGs published between January 1, 2020, and December 31, 2020, which were written in English or Chinese. A search strategy using the keywords “Coronavirus,” “COVID-19,” “SARS-CoV-2,” “severe acute respiratory syndrome coronavirus 2,” “guideline,” “guidance,” and “recommendation” was employed (full search strategies are presented in Appendix A).

### 2.2. Inclusion Criteria and Exclusion Criteria

We included CPGs for the diagnosis, treatment, care, rehabilitation, and management of COVID-19 and their complications. We included both CPGs published in journals and on websites.

The following guidelines were excluded: (1) guidelines exclusively concerning prevention, control of infection, screening, surveillance, or mental health, for most recommendations or measurements in these guidelines are not specific to COVID-19; (2) guidelines on other diseases covering COVID-19 as comorbidity, for those guidelines are not totally on COVID-19 patients; (3) translations, interpretations, and protocols of guidelines, for they are not CPGs or original versions of guidelines; (4) earlier versions of guidelines, for which an updated version is available, and repeated publications or editions of the same guideline; and (5) expert consensus statements, for they are not CPGs.

### 2.3. Guideline Selection and Data Extraction

Search results were imported into an EndNote library, and duplicates were identified. Each study was screened by two independent reviewers. The final inclusion of CPGs was agreed on by consensus. Disagreements were solved by consultation with a third author. We developed a purpose-designed spreadsheet for data extraction of the basic characteristics. Extracted characteristics included the following: title, country/organization of publication, month of publication, developer, version (original or updated), evidence-based development, interim CPG (yes/no) (interim guidelines are produced when the available data and information are most certainly incomplete, especially if additional data are anticipated in the near future; they usually have a very focused scope and a short shelf-life [54]), format of publication (published in a journal or on a website), rating system for the quality of evidence and strength of recommendations, methods to reach consensus, funding, conflicts of interest, and fulfilling the definition and criteria made by the IOM in 2011 (yes/no) [55]. And this definition is the most authoritative definition of evidence-based clinical practice guidelines available now [56].

### 2.4. Quality Appraisal of Guidelines

The Appraisal of Guidelines for Research & Evaluation II (AGREE II) [57] instrument, which contains 23 items grouped into six main domains, was used to assess the methodological quality of CPGs. The explanation of the six domains and 23 items of AGREE II is shown in Table 1. We appraised all COVID-19 CPGs according to the AGREE II User's Manual [57]. Eighteen reviewers in six groups of three (Group 1: S Zhao, J Cao, and S Wu; Group 2: Z Wang, Q Shi, and Q Guo; Group 3: S Lu, H Zhang, and L Wang; Group 4: H Liu, J Zhang, and H Lan; Group 5: M Ren, Y Liu, and P Wang; and Group 6: Q Zhou, Q Li, and X Zhang) evaluated all CPGs. The score for each domain (domain score (DS)) was obtained by summing and normalizing all individual item scores in this domain. Examples of the calculation method of a domain score and the overall domain score (overall score (OS)) for six domains of a CPG for AGREE II are shown in Table 2. We also reported the mean DS and OS over all CPGs.

The Reporting Items for practice Guidelines in HealThcare (RIGHT) checklist [58], which contains 35 items grouped into seven domains, was used to examine the reporting quality of CPGs. The explanation of the seven domains and 35 items of RIGHT is shown in Table 3. The CPGs were divided into seven pairs of reviewers (Group 1: S Zhao and J Cao; Group 2: Z Wang and Q Shi; Group 3: S Lu and H Zhang; Group 4: H Liu and J Zhang; Group 5: L Wang and X Zhang; Group 6: M Ren and Y Liu; and Group 7: S Wu and Q Guo) for appraisal. Each item was evaluated by both reviewers independently as either “reported” or “not reported.” An example of how to calculate the domain reporting rate (DRR) and the seven overall reporting rates (ORR) for a CPG is shown in Table 4. We also reported the mean DRR and ORR over all CPGs.

### 2.5. Analysis of the Recommendations and Evidence Used to Make Recommendations

For the six countries or organizations with the most published CPGs, we identified the recommendations regarding remdesivir, glucocorticoids, hydroxychloroquine/chloroquine, interferon, and lopinavir-ritonavir in COVID-19 CPGs published by the six countries or organizations. There was already high-quality evidence (It refers to the randomized controlled trials and systematic reviews of which the results were based on direct data from COVID-19 patients) that could prove the effectiveness of those treatments in treating COVID-19 patients. And we thought the evidence could support guidelines to make recommendations consistent. Four reviewers (S Zhao, S Lu, Z Wang, and H Liu) independently accessed the direction of recommendations on those treatments in COVID-19 CPGs. For each treatment or therapy, the direction of recommendations in each CPG was assessed as for if the source recommended the intervention in the treatment of COVID-19 or if the source did not recommend the intervention but declared the intervention could be considered in certain clinical situations. And the direction of recommendations in each CPG was assessed as against if the source did not recommend the intervention (including developers of CPGs did not recommend for or against the intervention due to insufficient evidence, and the source recommended that the intervention should only be used in clinical trials). Then, we also extracted the evidence (randomized controlled trials and systematic reviews) used to make recommendations in the CPGs if available.

### 2.6. Statistical Analysis and Quality Control

All reviewers had experience in using AGREE II and RIGHT to evaluate CPGs. The agreement among the AGREE II scores of the reviewers within each domain was measured by the intraclass correlation coefficient (ICC) to assess the reliability. The IBM SPSS Statistics 25.0 software was used to calculate the ICC value. We used Microsoft Excel 2019 to calculate the AGREE II DS and the RIGHT DRR. We compared the effect sizes between the categories of selected dichotomous characteristics (evidence-based process, version (original or updated), interim CPG (yes/no), format of publication (published in a journal or on a website), rating system for the quality of evidence and strength of recommendations, funding, and conflicts of interest) with the mean difference (MD) and 95% confidence interval (CI) using the RevMan 5.3 software.

## 3. Results

### 3.1. Search Results

**Figure 1:**
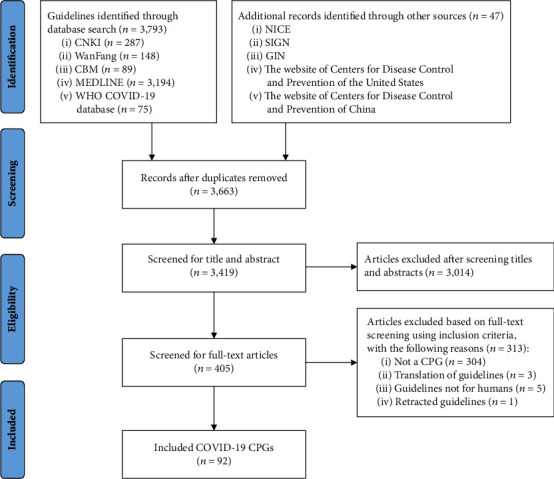
Flow diagram of the identification process for clinical practice guidelines on COVID-19.

The literature search yielded 3,840 records. After excluding irrelevant records, 92 CPGs were included. The process of guideline selection is illustrated in Figure 1, and the details of the selected guidelines are reported in Appendix B.

### 3.2. Characteristics of COVID-19 CPGs

The 92 COVID-19 CPGs were published by 19 countries or multinational organizations. Only seven (7.6%) met the IOM definition of CPGs, essentially meaning that their recommendations were informed by a systematic review of evidence (Table 5). In the seven CPGs, three are living guidelines and three are rapid guidelines.

China was the country that developed the most COVID-19 CPGs (*n* = 29, 31.2%), of which only one (1.1%) met the criteria of IOM [62]. Twenty (21.7%) of CPGs reported managing conflicts of interest. Sixty-seven (72.8%) CPGs did not use any system to rate the quality of evidence and strength of recommendations, and 66 (71.7%) CPGs did not report the method of how consensus for recommendations was reached. The highest numbers of COVID-19 CPGs were published in March (*n* = 19, 20.7%), April (*n* = 16, 17.4%), and May (*n* = 9, 9.8%). The characteristics of the included CPGs are provided in Table 6.

### 3.3. The Methodological Quality of COVID-19 CPGs

The overall agreement among reviewers for the evaluation with the AGREE II instrument was good (ICC = 0.801) [66, 67].

The mean AGREE II OS over all COVID-19 CPGs was 30.4%. The DS of all domains were below 50% (Table 7). Rigor of development had the lowest DS (19.2%), and clarity of presentation had the highest DS (44.7%).

**Figure 2:**
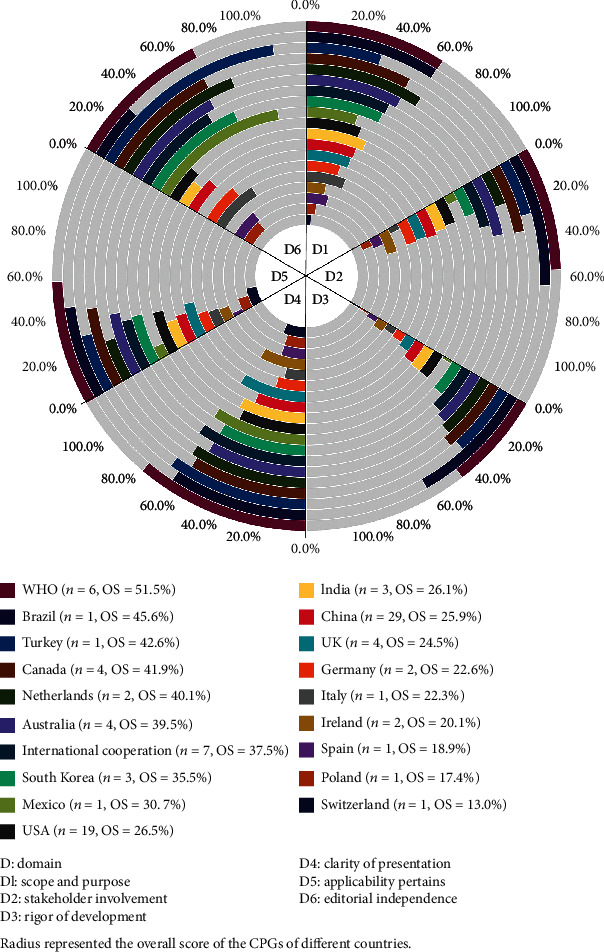
The AGREE II domain scores and overall scores (OS) of COVID-19 CPGs by developer country or organization.

We analyzed the CPGs' methodological quality according to different countries or organizations (Figure 2). The AGREE II scores varied greatly across CPGs produced by different countries or organizations. CPGs developed by WHO (*n* = 6) got the highest OS (51.5%), but the DS of WHO CPGs were highest in only two domains (clarity of presentation, DS = 67.0%; applicability pertains, DS = 47.9%). CPGs developed in Brazil had the highest scores in stakeholder involvement (DS = 53.7%) and rigor of development (DS = 50.7%). CPGs scoring highest in scope and purpose (DS = 54.6%) and editorial independence (DS = 86.1%) were produced by the Netherlands and Turkey, respectively.

**Figure 3:**
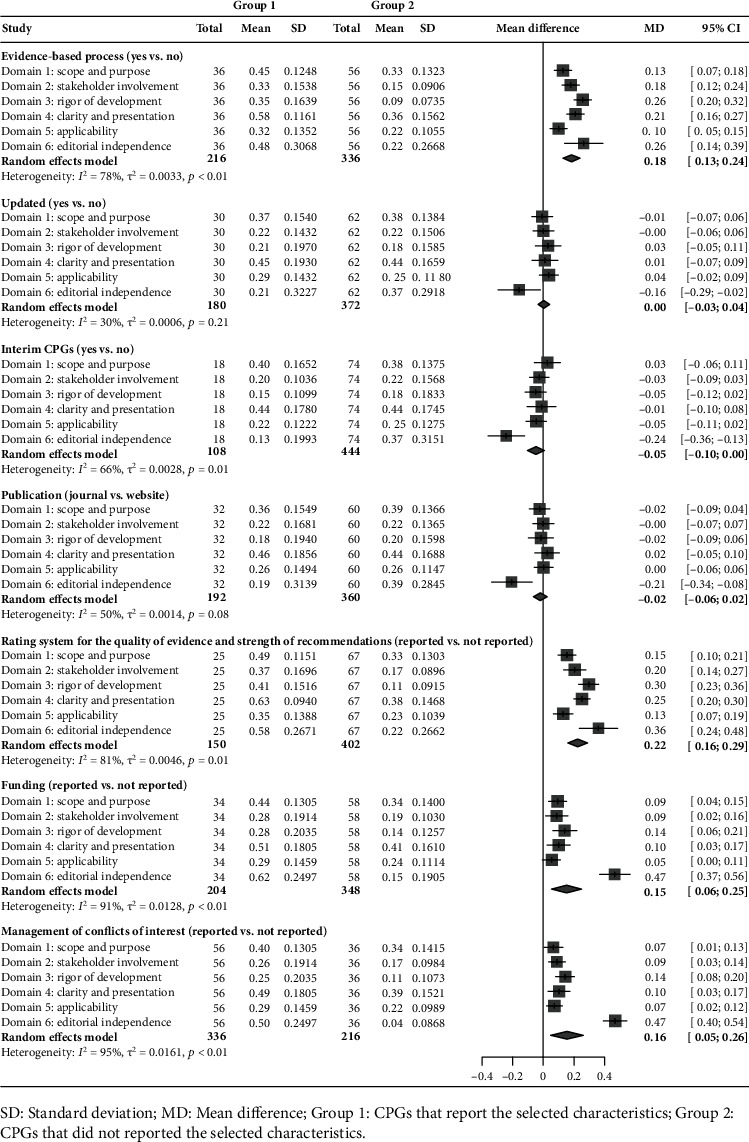
Comparison of methodological quality by selected characteristics.

Factors associated with better methodological quality included using the evidence-based development process (Mean difference [MD] = 0.18, 95% Confidence interval [CI] [0.13~0.24]), using a formal rating system for the quality of evidence and strength of recommendations (MD = 0.22, 95% CI [0.16~0.29]), reporting funding (MD = 0.15, 95% CI [0.06~0.25]), and managing conflicts of interest (MD = 0.16, 95% CI [0.05~0.26]) (Figure 3).

### 3.4. The Reporting Quality of COVID-19 CPGs

The mean ORR for all COVID-19 CPGs was 33.0%. The lowest DRR was in the domain “review and quality assurance” with a mean of 13.0%, and the highest was in the domain “basic information” with a mean of 51.4% (Table 7). Ten CPGs had a DRR below 50% in all seven domains.

**Figure 4:**
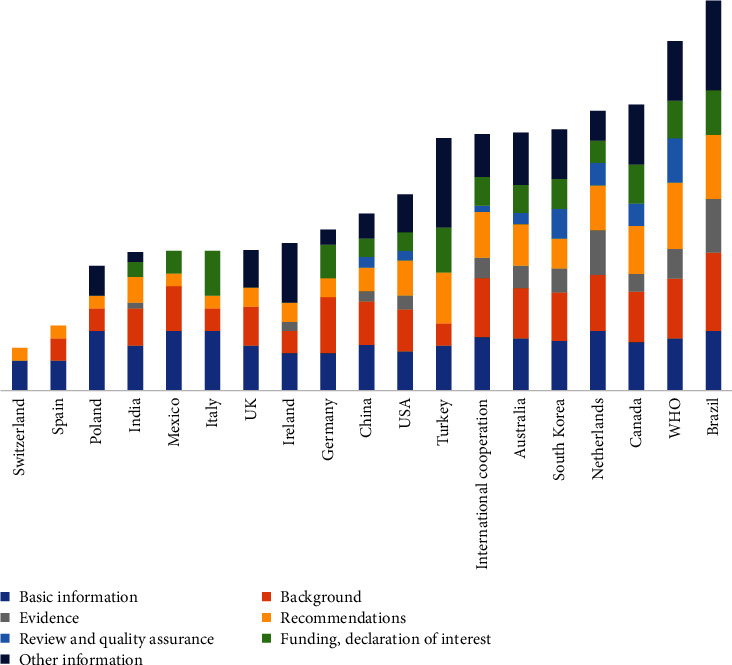
The RIGHT reporting rates by developer country or organization across COVID-19 clinical practice guidelines.

The RIGHT reporting rates varied greatly across CPGs developed by different countries and organizations (Figure 4). The CPG produced by Brazil had the highest ORR (68.8%), and the CPG developed in Switzerland had the lowest ORR (6.8%).

**Figure 5:**
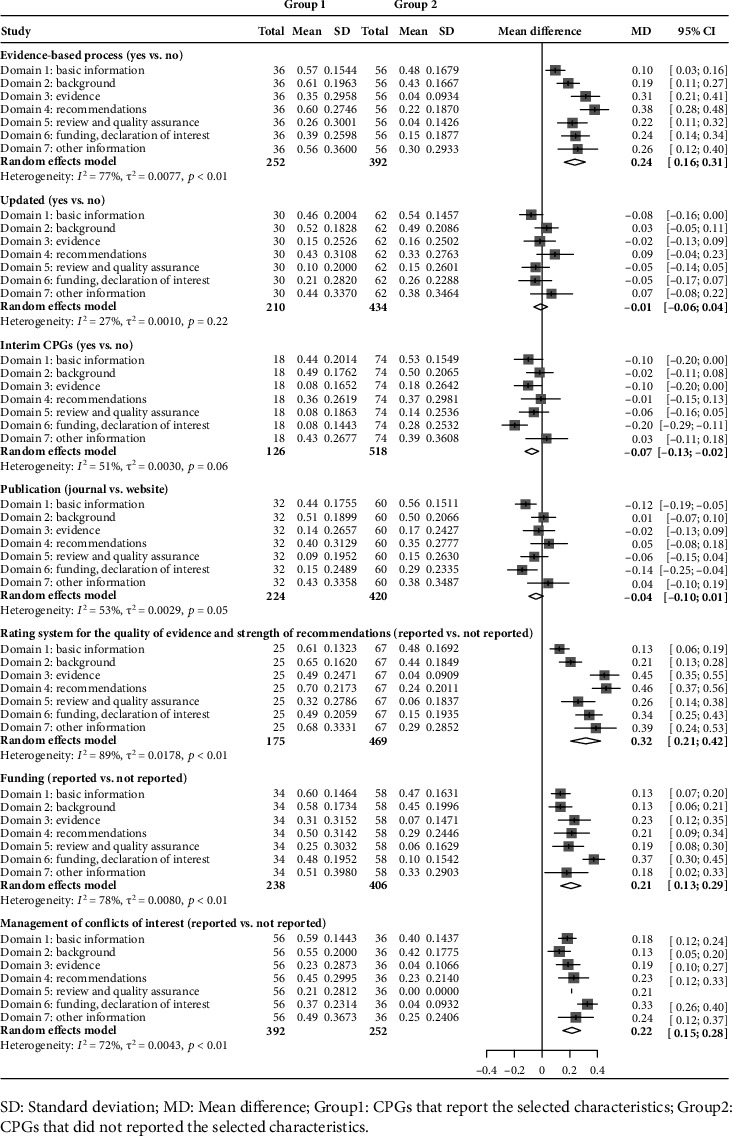
Comparison of reporting quality by selected characteristics.

CPGs that followed the factors, such as using the evidence-based development process (MD = 0.24, 95% CI [0.16~0.31]), using a formal rating system for the quality of evidence and strength of recommendations (MD = 0.32, 95% CI [0.21~0.42]), reporting funding (MD = 0.21, 95% CI [0.13~0.29]), and managing conflicts of interest (MD = 0.22, 95% CI [0.15~0.28]), were associated with higher reporting rates (Figure 5).

**Figure 6:**
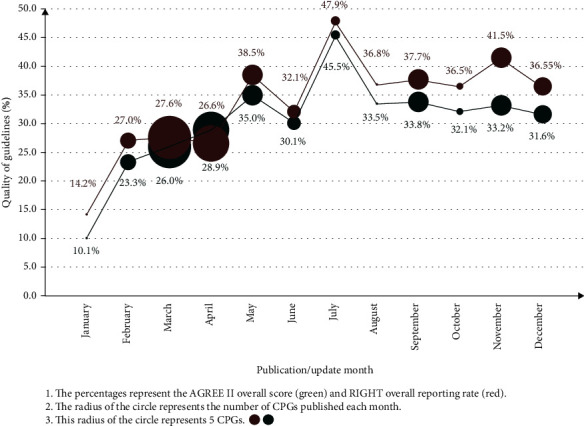
Temporal trends in the methodological and reporting qualities and quantities of COVID-19 clinical practice guidelines (CPGs).

The overall methodological quality and reporting quality had an increasing trend over time throughout the year 2020 (Figure 6).

### 3.5. Recommendations and Evidence on Some Treatments

**Figure 7:**
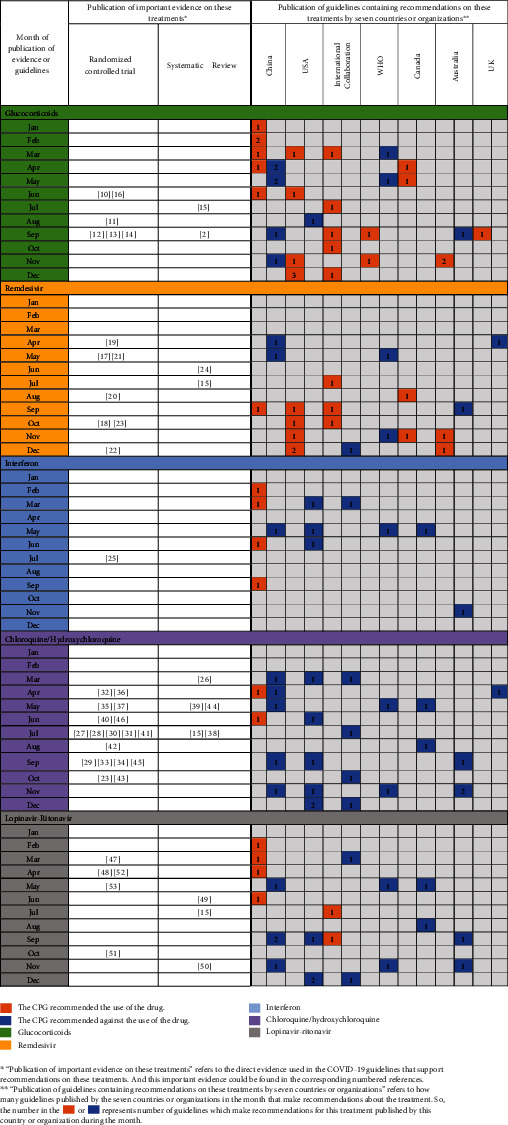
Evidence and recommendations on glucocorticoids, remdesivir, interferon, chloroquine/hydroxychloroquine, and lopinavir-ritonavir.

A total of 20 CPGs covered recommendations on remdesivir, 34 covered recommendations on glucocorticoids, 25 covered recommendations on hydroxychloroquine/chloroquine, 12 covered recommendations on interferon, and 21 covered recommendations on lopinavir-ritonavir, which were identified from six countries or organizations (Figure 7). Although randomized controlled trials and systematic reviews on the effectiveness of those treatments have been published, the recommendations still varied greatly across COVID-19 CPGs published in different countries or regions. Take glucocorticoids as an example; in June 2020, the research of the RECOVERY Collaborative Group had proved their effectiveness in the treatment of adult patients with COVID-19 [10], but some CPGs published in September and October still recommended against glucocorticoids, which may suggest that the CPGs do not make sufficient use of the latest evidence.

## 4. Discussion

Our study systematically searched for COVID-19 CPGs published in English and Chinese and found that the overall quality of the 92 identified COVID-19 CPGs was low. According to our findings, less than one-tenth of the CPGs met the new IOM definition of a CPG. A WHO rapid advice CPG [65] got the highest scores of methodological and reporting qualities of all included CPGs in our study, based on its high scores in methodological (OS = 72.8%) and reporting qualities (ORR = 83.8%). The evidence-based development process, funding, management of conflicts of interest, and rating systems for the quality of evidence and strength of recommendations were the main factors that impacted the quality of COVID-19 CPGs.

Dagens et al. [6] found that CPGs produced in the early stage of the COVID-19 pandemic had flaws in methodological quality, and no guidelines were based on evidence from systematic reviews. Our previous research [7] also found that both the methodological and reporting qualities of COVID-19 CPGs tended to be low. Luo et al. [9] found that the diagnosis and treatment recommendations between some COVID-19 guidelines are highly consistent when there was no effective treatment against COVID-19. Our results were consistent with some of the findings of these studies. We found that only a small percentage of the COVID-19 CPGs were informed by systematic reviews and used the GRADE approach. The overall quality of the CPGs still needs to be improved, but the methodological and reporting qualities of COVID-19 CPGs tended to improve consistently over time. There was conclusive evidence about some COVID-19 treatments (remdesivir, glucocorticoids, hydroxychloroquine/chloroquine, interferon, and lopinavir-ritonavir) [2, 10–53], but the consistency of recommendations in the COVID-19 CPGs was not good, which is different from the research results of Luo et al. [9].

Our study has several strengths. We comprehensively searched the literature from literature databases and websites and aimed to include all COVID-19 CPGs published in 2020. Our analysis could thus clearly identify how the quantity and quality of COVID-19 CPGs have changed over the year. In addition, we analyzed why the quality of COVID-19 CPGs was low. We also evaluated both the methodological and reporting qualities of included 92 COVID-19 CPGs. Most importantly, we systematically evaluated the consistency of recommendations and published evidence for the treatment of remdesivir, glucocorticoids, hydroxychloroquine/chloroquine, interferon, and lopinavir-ritonavir in COVID-19 CPGs. To our knowledge, this is the first review of those treatments in COVID-19 CPGs' recommendations produced during 2020. However, our study also had some limitations. Firstly, we excluded guidelines not available in English or Chinese. Secondly, researchers from all over the world have also published hundreds of expert consensus documents of COVID-19, but we did not include these in our analysis. Finally, when we calculated the domain scores of AGREE II and RIGHT of CPGs, we considered all domains to have equal weight, although in practice some domains may be more important than others when determining the overall quality.

Our study provides some important lessons for CPG developers on how to develop evidence-based CPGs during a pandemic such as COVID-19. First, we found a large amount of substantially overlapping CPGs of COVID-19. This means that COVID-19 CPGs which covered the same clinical questions or recommendations were published, and a huge amount of waste of workload funding and other resources could be at least partly prevented by prior coordination and collaboration among developers. The excessive duplication of CPGs which covered the same recommendations has also created confusion for clinicians in the appropriate application of those guidelines. For example, the USA and China both produced dozens of low-quality CPGs for COVID-19. These guidelines covered overlapping clinical questions, but thedirection of recommendations of those CPGs varied, which is confusing for decision-making. One solution to this problem is to promote prospective registration of CPGs on the same platform from the beginning of the development, similar to clinical trials and systematic reviews [68]. Registering a CPG can reduce duplication development of guidelines on the same clinical questions, increase the transparency of the development process, and promote cooperation among guideline developers all over the world [69, 70]. Since the International Practice Guideline Registry Platform has been created, developers could register guidelines prospectively and internationally on this platform (http://www.guidelines-registry.org/?lang=zh_CN). Second, guidelines produced in an emergency should be considered “living” guidelines with a totally transparent development process [6]. COVID-19 has rapidly become a disease associated with unbridled uncertainty. To respond to the uncertainties around the disease, CPGs should be updated and strengthened as evidence evolves [3]. The Guidelines International Network (GIN) also suggested that guideline recommendations should be clear evidence-based statements that aim to provide guideline users with clear directions for effective delivery of care [71]. So, it is necessary to develop a living guideline for it can facilitate the timely translation of the latest research findings to clinical practice. Third, the key messages of most of the guidelines were not fully and transparently reported in the full text. If developers strictly follow rigorous standards for developing trustworthy clinical practice guidelines such as those by the Institute of Medicine (US) Committee [55], the RIGHT checklist [58], and the GIN-McMaster Guideline Development Checklist [71], which are all supported by international guideline organizations, researchers then can better assess the quality of CPGs and users could better understand and apply CPGs.

## 5. Conclusion

Both the methodological and reporting qualities of COVID-19 CPGs increased over time, but there is still room for further improvement. The lack of effective use of available evidence and management of conflicts of interest were the main reasons for the low quality of the CPGs. The use of formal rating systems for the quality of evidence and strength of recommendations may help to improve the quality of CPGs in the context of the COVID-19 pandemic. During the pandemic, we suggest developing a living guideline of which recommendations are supported by a systematic review for it can facilitate the timely translation of the latest research findings to clinical practice. We also suggest that CPG developers should register the guidelines in a registration platform at the beginning for it can reduce duplication development of guidelines on the same clinical topic, increase the transparency of the development process, and promote cooperation among guideline developers all over the world. Since the International Practice Guideline Registry Platform has been created, developers could register guidelines prospectively and internationally on this platform.

## Figures and Tables

**Table 1:** Description of the AGREE II domains.

	Description of the AGREE II domains
Domain 1	Scope and Purpose is concerned with the overall aim of the guideline, the specific health questions, and the target population (items 1-3).
Domain 2	Stakeholder Involvement focuses on the extent to which the guideline was developed by the appropriate stakeholders and represents the views of its intended users (items 4-6).
Domain 3	Rigor of Development relates to the process used to gather and synthesize the evidence and the methods to formulate the recommendations and to update them (items 7-14).
Domain 4	Clarity of Presentation deals with the language, structure, and format of the guideline (items 15-17).
Domain 5	Applicability pertains to the likely barriers and facilitators to implementation, strategies to improve uptake, and resource implications of applying the guideline (items 18-21).
Domain 6	Editorial Independence is concerned with the formulation of recommendations not being unduly biased with competing interests (items 22-23).

**Table 2:** AGREE II domain score calculation example of three reviewers giving the following scores for Domain 1 (Scope and Purpose) for one guideline.

	Item 1	Item 2	Item 3	Total
Reviewer 1	5	6	6	17
Reviewer 2	6	6	7	19
Reviewer 3	2	4	3	9
Total obtained score	13	16	16	45

**M** **a** **x** **i** **m** **u** **m** **possible** **score** = 7 (**strongly** **agree**) × 3 (**items**) × 3 (**appraisers**) = 63				
**M** **i** **n** **i** **m** **u** **m** **possible** **score** = 1 (**strongly** **disagree**) × 3 (**items**) × 3 (**appraisers**) = 9				
$\text{The }scaled\;domain\;score=\frac{\left(Obtained\;score-Minimum\;possible\;score\right)}{\;\left(Maximum\;score-Minimum\;possible\;score\right)}\times 100\%=\frac{\left(45-9\right)}{\left(63-9\right)}\times 100\%=66.7\%$ $Mean\;score\;\text{of }Domain\;1=\frac{\Sigma \left(Scaled\;domain\;score\;\text{of }each\;individual\;guideline\right)}{\;Total\;number\;\text{of }guidelines\;}$				

**Table 3:** Description of the RIGHT domains.

	Description of the RIGHT domains
Domain 1	Basic information contains the information that need to be reported in the title/subtitle, executive summary, abbreviations and acronyms, and corresponding developer (items 1a-4).
Domain 2	In the background, the guideline authors should give a brief description of the health problem(s), report aim(s) of the guideline and specific objectives, target population, end users, and settings and guideline development groups (items 5-9b).
Domain 3	Evidence is about what should be reported on healthcare questions, systematic reviews, and assessment of the certainty of the body of evidence (items 10a-12).
Domain 4	Recommendations provide how to write recommendations, rationale/explanation for recommendations, and evidence to decision processes (items 13a-15).
Domain 5	The process of external review and quality assurance should be reported in the review and quality assurance (items 16-17).
Domain 6	Funding, declaration, and management of interests (items 18a-19b): (1) Funding source(s) and role(s) of the funder (2) Declaration and management of interest
Domain 7	Other information (items 20-22): (1) Describe where the guideline, its appendices, and other related documents can be accessed (2) Suggestions for further research (3) Limitations of the guideline

**Table 4:** RIGHT calculation example of the domain reporting rate from results by two reviewers in Domain 1 (basic information) for one guideline.

	Title/subtitle			Executive summary	Abbreviations and acronyms	Corresponding developer	Reported items	Total items
Items	1a	1b	1c	2	3	4	—	6
Reviewer 1	Reported	Reported	Reported	Reported	Reported	Reported		
Reviewer 2	Reported	Reported	Reported	Not	Reported	Reported		
Reviewer 3^∗^	—	—	—	Not	—	—		
Final result	Reported	Reported	Reported	Not	Reported	Reported	5	

$\text{Domain }1\text{ reporting rate}=\frac{\text{reported items}}{\text{total items}}\times 100\%=\frac{5}{6}\times 100\%=83.3\%$								

^∗^Third reviewer was called only to solve discrepancies.

**Table 5:** Clinical practice guidelines (CPGs) that met the Institute of Medicine (IOM) definition or criteria.

Developer country/ organization	CPGs that met IOM criteria							
	Establishing transparency	Management of conflict of interests	Guideline development group composition	Systematic review intersection	Establishing evidence foundations for and rating strength of recommendations	Articulation of recommendations	External review	Updating
Canada [59]	**√**	**√**	**√**	**√**	**√**	**√**	**√**	**√**
WHO [60]	**√**	**√**	**√**	**√**	**√**	**√**	**√**	**√**
WHO [61]	**√**	**√**	**√**	**√**	**√**	**√**	**√**	**√**
China [62]	**√**	**√**	**√**	**√**	**√**	**√**	**√**	**√**
Canada [63]	**×** ^−^	**√**	**√**	**√**	**√**	**√**	**√**	**√**
Australia [64]	**√**	**√**	**√**	**√**	**√**	**√**	**√**	**√**
WHO [65]	**√**	**√**	**√**	**√**	**√**	**√**	**√**	**√**

These CPGs did not report funding received.

**Table 6:** Characteristics of the clinical practice guidelines (CPGs) on COVID-19.

Characteristic		Number (*n* = 92)	Percentage (%)
Publication month	Jan	1	1.1
	Feb	7	7.6
	Mar	19	20.7
	Apr	16	17.4
	May	9	9.8
	Jun	6	6.5
	Jul	4	4.3
	Aug	1	1.1
	Sep	9	9.8
	Oct	3	3.3
	Nov	9	9.8
	Dec	8	8.6

Publication country/organization	China	29	31.5
	USA	19	20.7
	International cooperation (other than WHO)	7	7.6
	UK	4	4.3
	Canada	4	4.3
	Australia	4	4.3
	WHO	6	6.5
	India	3	3.3
	South Korea	3	3.3
	Netherlands	2	2.2
	Ireland	2	2.2
	Germany	2	2.2
	Turkey	1	1.1
	Switzerland	1	1.1
	Spain	1	1.1
	Poland	1	1.1
	Mexico	1	1.1
	Italy	1	1.1
	Brazil	1	1.1

Version of CPGs	Original	62	67.4
	Updated	30	32.6

Interim CPGs	Yes	18	19.6
	No	74	80.4

Publication format	Journal	60	65.2
	Website only	32	34.8

Developer	Society/association^∗^	42	45.2
	University	15	16.1
	National institution/council	10	10.8
	Hospital	12	12.9
	WHO	6	6.5
	National Centers for Disease Control (CDC)^∗^	6	6.5
	Not mentioned	2	2.2

Rating system for the quality of evidence and strength of recommendations	GRADE system	20	21.7
	Other systems	5	5.4
	Not reported	67	72.8

Methods to reach consensus	Reported	26	28.3
	Not reported	66	71.7

Funding	Reported funding	17	18.5
	Reported explicitly not to have received funding	17	18.5
	Not reported	58	63.0

Management of conflicts of interest	Yes	20	21.7
	No	36	39.1
	Not reported	36	39.1

^∗^One CPG that was codeveloped by a CDC and a society.

**Table 7:** The domain scores (DS) and overall score (OS) of the AGREE II instrument and the domain reporting rates (DRR) and overall reporting rates (ORR) of the RIGHT checklist over all COVID-19 clinical practice guidelines (CPGs).

	Domains	Score/reporting rate (%)	Number of CPGs in each quartile of the score			
		Mean ± SD^∗^	<25%	25%-50%	50%-75%	>75%
AGREE II	Scope and purpose	37.7% ± 14.4%	20 (21.7%)	54 (58.7%)	18 (19.6%)	0 (0.0%)
	Stakeholder involvement	22.3% ± 14.8%	59 (64.1%)	28 (30.4%)	4 (4.3%)	1 (1.1%)
	Rigor of development	19.2% ± 17.3%	65 (70.7%)	19 (20.7%)	8 (8.7%)	0 (0.0%)
	Clarity and presentation	44.7% ± 17.5%	14 (15.2%)	42 (45.7%)	32 (34.8%)	4 (4.3%)
	Applicability	26.1% ± 12.8%	50 (54.3%)	37 (40.2%)	5 (5.4%)	0 (0.0%)
	Editorial independence	32.1% ± 31.1%	44 (47.8%)	28 (30.4%)	5 (5.4%)	15 (16.3%)
	OS	30.4% ± 13.7%	40 (43.5%)	43 (46.7%)	9 (9.8%)	0 (0.0%)

RIGHT	Basic information	51.4% ± 17.0%	3 (3.3%)	58 (63.0%)	26 (28.3%)	5 (5.4%)
	Background	50.0% ± 20.1%	18 (19.6%)	40 (43.5%)	30 (32.6%)	4 (4.3%)
	Evidence	15.9% ± 25.1%	71 (77.2%)	10 (10.9%)	5 (5.4%)	6 (6.5%)
	Recommendations	36.5% ± 29.1%	34 (37.0%)	32 (34.8%)	14 (15.2%)	12 (13.0%)
	Review and quality assurance	13.0% ± 24.3%	70 (76.1%)	20 (21.7%)	0 (0.0%)	2 (2.2%)
	Funding, declaration of interest	24.2% ± 24.9%	62 (67.4%)	23 (25.0%)	7 (7.6%)	0 (0.0%)
	Other information	39.9% ± 34.5%	26 (28.3%)	38 (41.3%)	12 (13.0%)	16 (17.4%)
	ORR	33.0% ± 18.2%	38 (41.3%)	35 (38.0%)	18 (19.6%)	1 (1.1%)

^∗^SD: standard deviation.

## Appendix

### A. Search Strategy for MEDLINE

(#1) “COVID-19” (supplementary concept)
(#2) “severe acute respiratory syndrome coronavirus 2” (supplementary concept)
(#3) “Coronavirus” (mesh)
(#4) “Coronavirus Infections” (mesh)
(#5) “Coronavirus” (title/abstract)
(#6) “CoV” (title/abstract)
(#7) “HCoV” (title/abstract)
(#8) “ Human Coronaviruses” (title/abstract)
(#9) “2019-nCoV” (title/abstract)
(#10) “SARS-CoV-2” (title/abstract)
(#11) “severe acute respiratory syndrome coronavirus 2” (title/abstract)
(#12) “coronavirus disease-19” (title/abstract)
(#13) “2019 novel coronavirus disease” (title/abstract)
(#14) “COVID-19” (title/abstract)
(#15) “COVID19” (title/abstract)
(#16) OR/#1-#15
(#17) “Practice guideline” (publication type)
(#18) Guideline^∗^ (title/abstract)
(#19) Guidance^∗^ (title/abstract)
(#20) Recommendation^∗^ (title/abstract)
(#21) Statement^∗^ (title/abstract)
(#22) OR/#17-#21
(#23) #16 AND #22

### B. References of Included COVID-19 Guidelines

1. Organization, W., 2020. *Laboratory Testing For Coronavirus Disease 2019 (COVID-19) In Suspected Human Cases: Interim Guidance, 2 March 2020*. [online] Apps.who.int. Available at: https://apps.who.int/iris/handle/10665/331329 [Accessed 31 December 2020].

2. Organization, W., 2020. *Clinical Management Of Severe Acute Respiratory Infection (SARI) When COVID-19 Disease Is Suspected: Interim Guidance, 13 March 2020*. [online] Apps.who.int. Available at: https://apps.who.int/iris/handle/10665/331446 [Accessed 31 December 2020].

3. Cdc.gov. 2020. *Evaluating And Testing Persons For Coronavirus Disease 2019*. [online] Available at: https://www.cdc.gov/coronavirus/2019-nCoV/hcp/clinical-criteria.html [Accessed 31 December 2020].

4. Centers for Disease Control and Prevention. 2020. *Interim Laboratory Biosafety Guidelines For Handling And Processing Specimens Associated With Coronavirus Disease 2019 (COVID-19)*. [online] Available at: https://www.cdc.gov/coronavirus/2019-nCoV/lab/lab-biosafety-guidelines.html [Accessed 31 December 2020].

5. Centers for Disease Control and Prevention. 2020. *Interim Guidelines For Collecting, Handling, And Testing Clinical Specimens From Persons For Coronavirus Disease 2019 (COVID-19)*. [online] Available at: https://www.cdc.gov/coronavirus/2019-ncov/lab/guidelines-clinical-specimens.html?CDC_AA_refVal=https%3A%2F%2Fwww.cdc.gov%2Fcoronavirus%2F2019-ncov%2Fguidelines-clinical-specimens.html [Accessed 31 December 2020].

6. 陈韬,陈广,郭威,谢敏,马科,严丽,陈素华,冯玲.新型冠状病毒感染的肺炎诊疗快速指南(第三版)[J].医药导报,2020,39(03):305-307. Available at: https://kns.cnki.net/kcms/detail/detail.aspx?dbcode=CJFD&dbname=CJFDLAST2020&filename=YYDB202003007&v=stwY5YrKvb17FArVwlTzi7OJlMum68Ly2Ub2C%25mmd2FPgqNOgNnIyE70FjlqcaShFFSJk [Accessed 31 December 2020].

7. Centers for Disease Control and Prevention. 2020. ;*Interim Clinical Guidance For Management Of Patients With Confirmed Coronavirus Disease (COVID-19)*. [online] Available at: https://www.cdc.gov/coronavirus/2019-ncov/hcp/clinical-guidance-management-patients.html [Accessed 31 December 2020].

8. Med.china.com.cn. 2020. *新型冠状病毒感染的肺炎实验室检测技术指南*. [online] Available at: http://med.china.com.cn/content/pid/158678/tid/1026 [Accessed 31 December 2020].

9. Kns.cnki.net. 2020. *新型冠状病毒感染的肺炎影像学诊断指南(2020第一版)*. [online] Available at: https://kns.cnki.net/kcms/detail/detail.aspx?dbcode=CJFD&dbname=CJFDLAST2020&filename=YXXZ202001007&v=iFXw3AygAs9H8KdB82gk3ds2nmJkoHEuKRiEFZ231wzvWrFfqdETSDAo%25mmd2FLQtRrJi [Accessed 31 December 2020].

10. Kns.cnki.net. 2020. 成人重症新型冠状病毒肺炎患者气道管理推荐意见(试行). [online] Available at: https://kns.cnki.net/kcms/detail/detail.aspx?dbcode=CJFD&dbname=CJFDZHYX&filename=ZHYX202010003&v=a8tHzAYwongF4AWaPH8QerWiWExF4QuDGFcik0Yq%25mmd2BwDWcg69%25mmd2F6FrEH%25mmd2B6WB5L2pb1 [Accessed 31 December 2020].

11. Kns.cnki.net. 2020. *儿童2019冠状病毒病(COVID-19)诊疗指南(第二版)*. [online] Available at: https://kns.cnki.net/kcms/detail/detail.aspx?dbcode=CJFD&dbname=CJFDLAST2020&filename=ZJYB202002003&v=H%25mmd2FqZxB%25mmd2FaFfjDrd0YrQZLaHC4Q8du4BmMOsyZMW77j215KXb%25mmd2BM9da%25mmd2F8%25mmd2FDgEwRZS0m [Accessed 31 December 2020].

12. Wax, R. and Christian, M., 2020. Practical recommendations for critical care and anesthesiology teams caring for novel coronavirus (2019-nCoV) patients. *Canadian Journal of Anesthesia/Journal canadien d'anesthésie*, [online] 67(5), pp.568-576. Available at: https://link.springer.com/article/10.1007/s12630-020-01591-x [Accessed 31 December 2020].

13. Kns.cnki.net. 2020. *新型冠状病毒肺炎恢复期中医药综合干预方案专家指导意见(草案)*. [online] Available at: https://kns.cnki.net/kcms/detail/detail.aspx?dbcode=CJFD&dbname=CJFDLAST2020&filename=BJZO202002003&v=nk4FEwsUFBZsiQ1SYIj4M0hRYLhBcHebIYBkyR0TkpRmwvfD6x41bFu4%25mmd2Fkakcg2B [Accessed 31 December 2020].

14. Li, T., 2020. Diagnosis and clinical management of severe acute respiratory syndrome Coronavirus 2 (SARS-CoV-2) infection: an operational recommendation of Peking Union Medical College Hospital (V2.0). *Emerging Microbes & Infections*, 9(1), pp.582-585.

15. Isuog.org. 2020. *New ISUOG Interim Guidance - 2019 Novel Coronavirus Infection During Pregnancy And Puerperium: Information For Healthcare Professionals*. [online] Available at: https://www.isuog.org/resource/new-isuog-interim-guidance-2019-novel-coronavirus-infection-during-pregnancy-and-puerperium-information-for-healthcare-professionals.html [Accessed 31 December 2020].

16. Poston, J., Patel, B. and Davis, A., 2020. Management of Critically Ill Adults With COVID-19. *JAMA*,.

17. Chen, X., Liu, Y., Gong, Y., Guo, X., Zuo, M., Li, J., Shi, W., Li, H., Xu, X., Mi, W. and Huang, Y., 2020. Perioperative Management of Patients Infected with the Novel Coronavirus. *Anesthesiology*, 132(6), pp.1307-1316.

18. Kns.cnki.net. 2020. *新型冠状病毒肺炎针灸干预的指导意见(第二版)*. [online] Available at: https://kns.cnki.net/kcms/detail/detail.aspx?dbcode=CJFD&dbname=CJFDLAST2020&filename=ZGZE202005002&v=XRsQJ45w%25mmd2FRWHrxvJcJqcOzhLXRG%25mmd2B7z2ksLB6OfupgPVVHqZzoXhWdshbTEyBFkTJ [Accessed 31 December 2020].

19. Alhazzani, W., Møller, M., Arabi, Y., Loeb, M., Gong, M., Fan, E., Oczkowski, S., Levy, M., Derde, L., Dzierba, A., Du, B., Aboodi, M., Wunsch, H., Cecconi, M., Koh, Y., Chertow, D., Maitland, K., Alshamsi, F., Belley-Cote, E., Greco, M., Laundy, M., Morgan, J., Kesecioglu, J., McGeer, A., Mermel, L., Mammen, M., Alexander, P., Arrington, A., Centofanti, J., Citerio, G., Baw, B., Memish, Z., Hammond, N., Hayden, F., Evans, L. and Rhodes, A., 2020. Surviving Sepsis Campaign: Guidelines on the Management of Critically Ill Adults with Coronavirus Disease 2019 (COVID-19). *Critical Care Medicine*, 48(6), pp.e440-e469.

20. Cook, T., El‐Boghdadly, K., McGuire, B., McNarry, A., Patel, A. and Higgs, A., 2020. Consensus guidelines for managing the airway in patients with COVID ‐19. *Anaesthesia*, 75(6), pp.785-799.

21. Subject.med.wanfangdata.com.cn. 2020. *新型冠状病毒肺炎影像学辅助诊断指南*. [online] Available at: http://subject.med.wanfangdata.com.cn/UpLoad/Files/202003/7ef94e8f78bb4a0fa485c4561e386d8d.pdf [Accessed 31 December 2020].

22. Kns.cnki.net. 2020. *新型冠状病毒肺炎患者康复护理工作指导意见*. [online] Available at: https://kns.cnki.net/KCMS/detail/35.1329.R.20200408.1531.001.html?uid=WEEvREcwSlJHSldRa1FhcEFLUmViSFRpMTRZN2NiWkVLYmtpOHRaVTdVZz0=$9A4hF_YAuvQ5obgVAqNKPCYcEjKensW4IQMovwHtwkF4VYPoHbKxJw!!&v=MDc0OTNMRzRITkhNcTQ5Q1pPc1BZdzlNem1SbjZqNTdUM2ZscVdNMENMTDdSN3FkWitackZDL2xWcnJBSTFjPUl6VFRi [Accessed 31 December 2020].

23. Kns.cnki.net. 2020. *2019新型冠状病毒肺炎呼吸康复指导意见(第二版) - 中国知网*. [online] Available at: https://kns.cnki.net/kcms/detail/detail.aspx?dbcode=CJFD&dbname=CJFDZHYX&filename=ZHJH202004008&v=cuUHRFmzFixn2a5cNn%25mmd2B4qFglQtd86xw36YALqr4VRx%25mmd2FDtI0kp2nJjo10KTWica2T [Accessed 31 December 2020].

24. Kns.cnki.net. 2020. *新型冠状病毒肺炎患者功能恢复的中西医结合康复训练指导建议*. [online] Available at: https://kns.cnki.net/kcms/detail/detail.aspx?dbcode=CJFD&dbname=CJFDLAST2020&filename=SHZZ202003004&v=jVRPtukznYgt1V26VXS7Lv6iUCS6ztnovihaA9iV8%25mmd2BhUX35JW5qewxdmnCDW9LCL [Accessed 31 December 2020].

25. Kns.cnki.net. 2020. *新型冠状病毒肺炎出院患者中西医结合康复指导*. [online] Available at: https://kns.cnki.net/kcms/detail/detail.aspx?dbcode=CJFD&dbname=CJFDLAST2020&filename=FYXB202004001&v=aOrLFH6UAqtEXOAryvWdKvqzqAsQQQEypUaxrr1F3ntxIp%25mmd2FJv1Vy3N92KzjHq8kY [Accessed 31 December 2020].

26. Kns.cnki.net. 2020. *新型冠状病毒肺炎影像检查诊断与感染控制指导意见*. [online] Available at: https://kns.cnki.net/KCMS/detail/31.1700.R.20200309.2001.002.html?uid=WEEvREcwSlJHSldRa1FhcEFLUmViSFRpMTRUK1hnY0lsbEZlSFd6RTJNYz0=$9A4hF_YAuvQ5obgVAqNKPCYcEjKensW4IQMovwHtwkF4VYPoHbKxJw!!&v=MzEyNzU3UjdxZForWnJGQy9sVkx2Skkxcz1QQ2ZmZDdHNEhOSE1ySTlNWk9zUFl3OU16bVJuNmo1N1QzZmxxV00wQ0xM [Accessed 31 December 2020].

27. Emergency.cdc.gov. 2020. *Updated Guidance On Evaluating And Testing Persons For Coronavirus Disease 2019 (COVID-19)*. [online] Available at: https://emergency.cdc.gov/han/2020/HAN00429.asp [Accessed 31 December 2020].

28. Feng, Y., Ni, L., Jieying, H., Lulu, W., Guansheng, S., Nanshan, Z. and Zeguang, Z., 2020. *Pulmonary Rehabilitation Guidelines In The Principle Of 4S For Patients Infected With 2019 Novel Coronavirus (2019-Ncov)*. [online] Rs.yiigle.com. Available at: http://rs.yiigle.com/CN112147202003/1184456.htm [Accessed 31 December 2020].

29. Swiss Society Of Intensive Care Medicine. Recommendations for the admission of patients with COVID-19 to intensive care and intermediate care units (ICUs and IMCUs). *Swiss Med Wkly*. 2020;150:w20227. Published 2020 Mar 24. doi:10.4414/smw.2020.20227

30. 2020. *Interim Guidance For The Use Of Tocilizumab In The Management Of Patients Who Have Severe COVID-19 With Suspected Hyperinflammation [V3.0]*. [PDF] feidhmeannacht na seibhise slainte health service executive. Available at: https://www.hse.ie/eng/about/who/acute-hospitals-division/drugs-management-programme/interim-recommendations-for-the-use-of-tocilizumab-in-the-management-of-patients-who-have-severe-covid-19-with-suspected-hyperinflammation.pdf [Accessed 31 December 2020].

31. World Health Organization, 2020. Clinical management of severe acute respiratory infection (SARI) when COVID-19 disease is suspected. Interim guidance. *Pediatria i Medycyna Rodzinna*, 16(1), pp.9-26.

32. Poon, L., Yang, H., Kapur, A., Melamed, N., Dao, B., Divakar, H., McIntyre, H., Kihara, A., Ayres‐de‐Campos, D., Ferrazzi, E., Di Renzo, G. and Hod, M., 2020. Global interim guidance on coronavirus disease 2019 (COVID‐19) during pregnancy and puerperium from FIGO and allied partners: Information for healthcare professionals. *International Journal of Gynecology & Obstetrics*, 149(3), pp.273-286.

33. 2020. *INITIAL GUIDANCE: Management Of Infants Born To Mothers With COVID-19 Date Of Document: April 2, 2020*. [PDF] American Academy of Pediatrics Committee on Fetus and Newborn, Section on Neonatal Perinatal Medicine, and Committee on Infectious Diseases. Available at: https://www.cpd.utoronto.ca/wp-content/uploads/2020/04/COVID-19-Initial-Newborn-Guidance.pdf [Accessed 31 December 2020].

34.0 2020. *Interim Guidance For The Use Of Antiviral Therapy In The Clinical Management Of Acute Respiratory Infection With SARS-Cov-2 (COVID-19). [V2.0]*. [PDF] feidhmeannacht na seibhise slainte health service executive. Available at: https://www.hse.ie/eng/about/who/acute-hospitals-division/drugs-management-programme/specific-antiviral-therapy-in-the-clinical-management-of-acute-respiratory-infection-with-sars-cov-2-covid-19-.pdf [Accessed 31 December 2020].

35. COVID-19 Treatment Guidelines. 2020. *Guidelines Introduction | COVID-19 Treatment Guidelines*. [online] Available at: <https://covid19treatmentguidelines.nih.gov/introduction/> [Accessed 31 December 2020].

36. Ye, Z., Rochwerg, B., Wang, Y., Adhikari, N., Murthy, S., Lamontagne, F., Fowler, R., Qiu, H., Wei, L., Sang, L., Loeb, M., Shen, N., Huang, M., Jiang, Z., Arabi, Y., Colunga-Lozano, L., Jiang, L., Koh, Y., Liu, D., Liu, F., Phua, J., Shen, A., Huo, T., Du, B., Zhai, S. and Guyatt, G., 2020. Treatment of patients with nonsevere and severe coronavirus disease 2019: an evidence-based guideline. *Canadian Medical Association Journal*, 192(20), pp.E536-E545.

37. Update to living WHO guideline on drugs for covid-19. *BMJ*. 2020;371:m4475. Published 2020 Nov 19. doi:10.1136/bmj.m4475. [Accessed 31 December 2020].

38. Idsociety.org. 2020. *Infectious Diseases Society Of America Guidelines On The Diagnosis Of COVID-19*. [online] Available at: https://www.idsociety.org/practice-guideline/covid-19-guideline-diagnostics/ [Accessed 31 December 2020].

39. Idsociety.org. 2020. *COVID-19 Guideline, Part 1: Treatment And Management*. [online] Available at: https://www.idsociety.org/practice-guideline/covid-19-guideline-treatment-and-management/ [Accessed 31 December 2020].

40. 2020. *Massachusetts General Hospital (MGH) COVID-19 Treatment Guidance*. [PDF] MASSACHUSETTS GENERAL HOSPITAL, pp.1-24. Available at: https://www.massgeneral.org/assets/MGH/pdf/news/coronavirus/mass-general-COVID-19-treatment-guidance.pdf [Accessed 31 December 2020].

41. Who.int. 2020. *Corticosteroids For COVID-19: Living Guidance*. [online] Available at: https://www.who.int/publications/i/item/WHO-2019-nCoV-Corticosteroids-2020.1 [Accessed 31 December 2020].

42. Who.int. 2020. *Clinical Management Of COVID-19*. [online] Available at: https://www.who.int/publications/i/item/clinical-management-of-covid-19 [Accessed 31 December 2020].

43. Nice.org.uk. 2020. *Overview | COVID-19 Rapid Guideline: Critical Care In Adults | Guidance | NICE*. [online] Available at: https://www.nice.org.uk/guidance/ng159 [Accessed 31 December 2020].

44. Kns.cnki.net. 2020. *新型冠状病毒肺炎恢复期中西医结合康复指南(第一版)*. [online] Available at: <https://kns.cnki.net/kcms/detail/detail.aspx?dbcode=CJFD&dbname=CJFDLAST2020&filename=TJZY202005003&v=eQYWW9KIc3Q2L6EIjfLgF8ebC3bTj99TguUl%25mmd2BXEkuIMeLSbZWfpOvGpoMy9USaEP> [Accessed 31 December 2020].

45. Bai, C., Chotirmall, S., Rello, J., Alba, G., Ginns, L., Krishnan, J., Rogers, R., Bendstrup, E., Burgel, P., Chalmers, J., Chua, A., Crothers, K., Duggal, A., Kim, Y., Laffey, J., Luna, C., Niederman, M., Raghu, G., Ramirez, J., Riera, J., Roca, O., Tamae-Kakazu, M., Torres, A., Watkins, R., Barrecheguren, M., Belliato, M., Chami, H., Chen, R., Cortes-Puentes, G., Delacruz, C., Hayes, M., Heunks, L., Holets, S., Hough, C., Jagpal, S., Jeon, K., Johkoh, T., Lee, M., Liebler, J., McElvaney, G., Moskowitz, A., Oeckler, R., Ojanguren, I., O'Regan, A., Pletz, M., Rhee, C., Schultz, M., Storti, E., Strange, C., Thomson, C., Torriani, F., Wang, X., Wuyts, W., Xu, T., Yang, D., Zhang, Z. and Wilson, K., 2020. Updated guidance on the management of COVID-19: from an American Thoracic Society/European Respiratory Society coordinated International Task Force (29 July 2020). *European Respiratory Review*, 29(157), p.200287.

46. Bartlett, R., Ogino, M., Brodie, D., McMullan, D., Lorusso, R., MacLaren, G., Stead, C., Rycus, P., Fraser, J., Belohlavek, J., Salazar, L., Mehta, Y., Raman, L. and Paden, M., 2020. Initial ELSO Guidance Document: ECMO for COVID-19 Patients with Severe Cardiopulmonary Failure. *ASAIO Journal*, 66(5), pp.472-474.

47. Bentley SK, Iavicoli L, Cherkas D, et al. Guidance and Patient Instructions for Proning and Repositioning of Awake, Nonintubated COVID-19 Patients. *Acad Emerg Med*. 2020;27(8):787-791. doi:10.1111/acem.14067

48. Chawla D, Chirla D, Dalwai S, et al. Perinatal-Neonatal Management of COVID-19 Infection - Guidelines of the Federation of Obstetric and Gynaecological Societies of India (FOGSI), National Neonatology Forum of India (NNF), and Indian Academy of Pediatrics (IAP). *Indian Pediatr*. 2020;57(6):536-548. doi:10.1007/s13312-020-1852-4

49. Chiotos K, Hayes M, Kimberlin DW, et al. Multicenter interim guidance on use of antivirals for children with COVID-19/SARS-CoV-2 [published online ahead of print, 2020 Sep 12]. *J Pediatric Infect Dis Soc*. 2020;piaa115. doi:10.1093/jpids/piaa115

50. Dulek DE, Fuhlbrigge RC, Tribble AC, et al. Multidisciplinary Guidance Regarding the Use of Immunomodulatory Therapies for Acute COVID-19 in Pediatric Patients [published online ahead of print, 2020 Aug 18]. *J Pediatric Infect Dis Soc*. 2020;piaa098. doi:10.1093/jpids/piaa098

51. Falavigna M, Colpani V, Stein C, et al. Guidelines for the pharmacological treatment of COVID-19. The task-force/consensus guideline of the Brazilian Association of Intensive Care Medicine, the Brazilian Society of Infectious Diseases and the Brazilian Society of Pulmonology and Tisiology. Diretrizes para o tratamento farmacológico da COVID-19. Consenso da Associação de Medicina Intensiva Brasileira, da Sociedade Brasileira de Infectologia e da Sociedade Brasileira de Pneumologia e Tisiologia. *Rev Bras Ter Intensiva*. 2020;32(2):166-196. doi:10.5935/0103-507x.20200039

52. Jin, Y., Zhan, Q., Peng, Z., Ren, X., Yin, X., Cai, L., Yuan, Y., Yue, J., Zhang, X., Yang, Q., Ji, J., Xia, J., Li, Y., Zhou, F., Gao, Y., Yu, Z., Xu, F., Tu, M., Tan, L., Yang, M., Chen, F., Zhang, X., Zeng, M., Zhu, Y., Liu, X., Yang, J., Zhao, D., Ding, Y., Hou, N., Wang, F., Chen, H., Zhang, Y., Li, W., Chen, W., Shi, Y., Yang, X., Wang, X., Zhong, Y., Zhao, M., Li, B., Ma, L., Zi, H., Wang, N., Wang, Y., Yu, S., Li, L., Huang, Q., Weng, H., Ren, X., Luo, L., Fan, M., Huang, D., Xue, H., Yu, L., Gao, J., Deng, T., Zeng, X., Li, H., Cheng, Z., Yao, X. and Wang, X., 2020. Chemoprophylaxis, diagnosis, treatments, and discharge management of COVID-19: An evidence-based clinical practice guideline (updated version). *Military Medical Research*, 7(1).

53. Kim SB, Huh K, Heo JY, et al. Interim Guidelines on Antiviral Therapy for COVID-19. *Infect Chemother*. 2020;52(2):281-304. doi:10.3947/ic.2020.52.2.281

54. Kurtaiş Aytür Y, Köseoğlu BF, Özyemişçi Taşkıran Ö, et al. Pulmonary rehabilitation principles in SARS-COV-2 infection (COVID-19): A guideline for the acute and subacute rehabilitation. *Turk J Phys Med Rehabil*. 2020;66(2):104-120. Published 2020 May 12. doi:10.5606/tftrd.2020.6444

55. Lee BJ, Lee JA, Kim KI, Choi JY, Jung HJ. A consensus guideline of herbal medicine for coronavirus disease 2019. *Integr Med Res*. 2020;9(3):100470. doi:10.1016/j.imr.2020.100470

56. Liu E, Smyth RL, Luo Z, et al. Rapid advice guidelines for management of children with COVID-19 [published correction appears in Ann Transl Med. 2020 Jun;8(12):807]. *Ann Transl Med*. 2020;8(10):617. doi:10.21037/atm-20-3754

57. Qu JM, Wang C, Cao B; Chinese Thoracic Society and Chinese Association of Chest Physicians. Guidance for the management of adult patients with coronavirus disease 2019. *Chin Med J (Engl)*. 2020;133(13):1575-1594. doi:10.1097/CM9.0000000000000899

58. Rochwerg B, Agarwal A, Zeng L, et al. Remdesivir for severe covid-19: a clinical practice guideline [published correction appears in BMJ. 2020 Nov 23;371:m4542]. *BMJ*. 2020;370:m2924. Published 2020 Jul 30. doi:10.1136/bmj.m2924

59. Sharma SK, Nuttall C, Kalyani V; Hemlata . Clinical nursing care guidance for management of patient with COVID-19. *J Pak Med Assoc*. 2020;70(Suppl 3)(5):S118-S123. doi:10.5455/JPMA.29

60. Sieswerda E, de Boer MGJ, Bonten MMJ, et al. Recommendations for antibacterial therapy in adults with COVID-19 - an evidence based guideline. *Clin Microbiol Infect*. 2020;27(1):61-66. doi:10.1016/j.cmi.2020.09.041

61. Spruit MA, Holland AE, Singh SJ, Tonia T, Wilson KC, Troosters T. COVID-19: Interim Guidance on Rehabilitation in the Hospital and Post-Hospital Phase from a European Respiratory Society and American Thoracic Society-coordinated International Task Force [published online ahead of print, 2020 Aug 13]. *Eur Respir J*. 2020;56(6):2002197. doi:10.1183/13993003.02197-2020

62. Kns.cnki.net. 2020. *新型冠状病毒肺炎(COVID-19)中西医结合临床诊疗快速建议指南*. [online] Available at: https://kns.cnki.net/kcms/detail/detail.aspx?dbcode=CJFD&dbname=CJFDLAST2020&filename=YJXU202002013&v=IFNWuzxuYr7zWjsa%25mmd2BN1F1GGjmGTOMFAJdC6A%25mmd2F9SF61Q2UqC%25mmd2Ba%25mmd2F1jjWIBGA352UtV [Accessed 31 December 2020].

63. Liang N, Ma Y, Wang J, et al. Traditional Chinese Medicine guidelines for coronavirus disease 2019. *J Tradit Chin Med*. 2020;40(6):891-896. doi:10.19852/j.cnki.jtcm.20200902.001

64. Health.nsw.gov.au. 2020. *NSW Health Interim Guidance On Use Of Antiviral And Immunomodulation Therapy In COVID-19 - Communities Of Practice*. [online] Available at: https://www.health.nsw.gov.au/Infectious/covid-19/communities-of-practice/Pages/guide-antiviral-therapy.aspx#document [Accessed 31 December 2020].

65. App.magicapp.org. 2020. *Magicapp - Making GRADE The Irresistible Choice - Guidelines And Evidence Summaries*. [online] Available at: https://app.magicapp.org/#/guideline/4632 [Accessed 31 December 2020].

66. Chen Q, Wang L, Yu W, et al. Recommendations for the prevention and treatment of the novel coronavirus pneumonia in the elderly in China. *Aging Med (Milton)*. 2020;3(2):66-73. Published 2020 Jun 9. doi:10.1002/agm2.12113

67. Rimensberger PC, Kneyber MCJ, Deep A, et al. Caring for Critically Ill Children With Suspected or Proven Coronavirus Disease 2019 Infection: Recommendations by the Scientific Sections' Collaborative of the European Society of Pediatric and Neonatal Intensive Care. *Pediatr Crit Care Med*. 2020;22(1):56-67. doi:10.1097/PCC.0000000000002599

68. RCPCH. 2020. *COVID-19 - Guidance For Management Of Children Admitted To Hospital*. [online] Available at: https://www.rcpch.ac.uk/resources/covid-19-guidance-management-children-admitted-hospital [Accessed 31 December 2020].

69. Kache S, Chisti MJ, Gumbo F, et al. COVID-19 PICU guidelines: for high- and limited-resource settings. *Pediatr Res*. 2020;88(5):705-716. doi:10.1038/s41390-020-1053-9

70. Peeters LM, Parciak T, Walton C, et al. COVID-19 in people with multiple sclerosis: A global data sharing initiative. *Mult Scler*. 2020;26(10):1157-1162. doi:10.1177/1352458520941485

71. Kluge S, Janssens U, Welte T, Weber-Carstens S, Marx G, Karagiannidis C. German recommendations for critically ill patients with COVID‑19. Empfehlungen zur intensivmedizinischen Therapie von Patienten mit COVID‑19. *Med Klin Intensivmed Notfmed*. 2020;115(Suppl 3):111-114. doi:10.1007/s00063-020-00689-w

72. 2020. *Guidelines On Clinical Management Of COVID – 19*. [PDF] Government of India Ministry of Health & Family Welfare Directorate General of Health Services (EMR Division), p.15. Available at: <https://www.mohfw.gov.in/pdf/GuidelinesonClinicalManagementofCOVID1912020.pdf> [Accessed 31 December 2020].

73. Mp.pl. 2020. *Management Of SARS-Cov-2 Infection: Recommendations Of The Polish Association Of Epidemiologists And Infectiologists As Of March 31, 2020 - Polish Archives Of Internal Medicine*. [online] Available at: https://www.mp.pl/paim/issue/article/15270 [Accessed 31 December 2020].

74. Thomas P, Baldwin C, Bissett B, et al. Physiotherapy management for COVID-19 in the acute hospital setting: clinical practice recommendations. *J Physiother*. 2020;66(2):73-82. doi:10.1016/j.jphys.2020.03.011

75. Felten-Barentsz KM, van Oorsouw R, Klooster E, et al. Recommendations for Hospital-Based Physical Therapists Managing Patients With COVID-19. *Phys Ther*. 2020;100(9):1444-1457. doi:10.1093/ptj/pzaa114

76. Zhao HM, Xie YX, Wang C; Chinese Association of Rehabilitation Medicine; Respiratory Rehabilitation Committee of Chinese Association of Rehabilitation Medicine; Cardiopulmonary Rehabilitation Group of Chinese Society of Physical Medicine and Rehabilitation. Recommendations for respiratory rehabilitation in adults with coronavirus disease 2019. *Chin Med J (Engl)*. 2020;133(13):1595-1602. doi:10.1097/CM9.0000000000000848

77. Fang F, Chen Y, Zhao D, et al. Recommendations for the Diagnosis, Prevention, and Control of Coronavirus Disease-19 in Children-The Chinese Perspectives. *Front Pediatr*. 2020;8:553394. Published 2020 Nov 5. doi:10.3389/fped.2020.553394

78. Ñamendys-Silva SA, Cherit GD. Recommendations for the management of critically ill adult patients with COVID-19. Recomendaciones de tratamiento para pacientes adultos graves con COVID-19. *Gac Med Mex*. 2020;156(3):246-248. doi:10.24875/GMM.M20000375

79. Calvo C, López-Hortelano MG, Vicente JCC, Martínez JLV; Grupo de trabajo de la Asociación Española de Pediatría para el brote de infección por Coronavirus, colaboradores con el Ministerio de Sanidad. Recommendations on the clinical management of the COVID-19 infection by the «new coronavirus» SARS-CoV2. Spanish Paediatric Association working group. *An Pediatr (Engl Ed)*. 2020;92(4):241.e1-241.e11. doi:10.1016/j.anpede.2020.02.002

80. 2020. *SARS Cov-2 Guidance Document*. [PDF] American Association for Respiratory Care, p.10. Available at: https://www.aarc.org/wp-content/uploads/2020/03/guidance-document-SARS-COVID19.pdf [Accessed 31 December 2020].

81. Köstenberger M, Hasibeder W, Dankl D, et al. SARS-CoV-2: recommendations for treatment in intensive care medicine. *Wien Klin Wochenschr*. 2020;132(21-22):664-670. doi:10.1007/s00508-020-01734-6

82. Sorbello M, El-Boghdadly K, Di Giacinto I, et al. The Italian coronavirus disease 2019 outbreak: recommendations from clinical practice. *Anaesthesia*. 2020;75(6):724-732. doi:10.1111/anae.15049

83. Akl EA, Blazic I, Yaacoub S, et al. Use of Chest Imaging in the Diagnosis and Management of COVID-19: A WHO Rapid Advice Guide [published online ahead of print, 2020 Jul 30]. *Radiology*. 2020;203173. doi:10.1148/radiol.2020203173

84. Kns.cnki.net. 2020. *危重症新型冠状病毒肺炎患者后ICU综合征呼吸康复推荐意见*. [online] Available at: <https://kns.cnki.net/kcms/detail/detail.aspx?dbcode=CJFD&dbname=CJFDZHYX&filename=ZHJH202009013&v=cuUHRFmzFiyz85M1BhYR%25mmd2FeLqU0Ex3WIj2V4G2RzIRRVxlWnIwjJQe3yWz%25mmd2FQ5sYZd> [Accessed 31 December 2020].

85. http://journal03.magtech.org.cn/. 2020. *新型冠状病毒肺炎影像诊断指南(2020年第二版简版)*. [online] Available at: http://journal03.magtech.org.cn/Jweb_sdykdxxb/CN/10.3969/j.issn.1006-7795.2020.02.004 [Accessed 31 December 2020].

86. Chen ZM, Fu JF, Shu Q, et al. Diagnosis and treatment recommendations for pediatric respiratory infection caused by the 2019 novel coronavirus. *World J Pediatr*. 2020;16(3):240-246. doi:10.1007/s12519-020-00345-5

87. Hong KH, Lee SW, Kim TS, et al. Guidelines for Laboratory Diagnosis of Coronavirus Disease 2019 (COVID-19) in Korea. *Ann Lab Med*. 2020;40(5):351-360. doi:10.3343/alm.2020.40.5.351

88. Api O, Sen C, Debska M, et al. Clinical management of coronavirus disease 2019 (COVID-19) in pregnancy: recommendations of WAPM-World Association of Perinatal Medicine. *J Perinat Med*. 2020;48(9):857-866. doi:10.1515/jpm-2020-0265

89. Kluge S, Janssens U, Welte T, et al. Recommendations for treatment of critically ill patients with COVID-19 : Version 3 S1 guideline [published online ahead of print, 2020 Nov 27]. Empfehlungen für die Behandlung von kritisch kranken Patienten mit COVID-19 : Version 3 S1-Leitlinie [published online ahead of print, 2020 Nov 27]. *Anaesthesist*. 2020;1-11. doi:10.1007/s00101-020-00879-3

90. Shetty VU, Brotherton BJ, Achilleos A, et al. Pragmatic Recommendations for Therapeutics of Hospitalized COVID-19 Patients in Low- and Middle-Income Countries [published online ahead of print, 2020 Dec 29]. *Am J Trop Med Hyg*. 2020;10.4269/ajtmh.20-1106. doi:10.4269/ajtmh.20-1106

91. Nice.org.uk. 2020. *Overview | COVID-19 Rapid Guideline: Managing The Long-Term Effects Of COVID-19 | Guidance | NICE*. [online] Available at: https://www.nice.org.uk/guidance/ng188 [Accessed 31 December 2020].

92. Henderson LA, Canna SW, Friedman KG, et al. American College of Rheumatology Clinical Guidance for Pediatric Patients with Multisystem Inflammatory Syndrome in Children (MIS-C) Associated with SARS-CoV-2 and Hyperinflammation in COVID-19. Version 2 [published online ahead of print, 2020 Dec 5]. *Arthritis Rheumatol*. 2020;10.1002/art.41616. doi:10.1002/art.41616
